# HIV-1 increases TLR responses in human primary astrocytes

**DOI:** 10.1038/srep17887

**Published:** 2015-12-16

**Authors:** M Jesús Serramía, M Ángeles Muñoz-Fernández, Susana Álvarez

**Affiliations:** 1 Laboratorio Inmuno-Biología Molecular, Hospital General Universitario Gregorio Marañón, Madrid, Spain; 2 Instituto de Investigación Sanitaria Gregorio Marañón (IISGM), Madrid, Spain; 3 Networking Research Center on Bioengineering, Biomaterials and Nanomedicine (CIBER-BBN), Madrid, Spain

## Abstract

Astrocytes are the major glial cell within the central nervous system and have a number of important physiological properties related to brain homeostasis. They provide trophic support to neurons and are immune cells with key roles during states-of-inflammation. The potential for production of proinflammatory cytokines and its consequences has been studied in the context of HIV-1 infection of normal human astrocytes (NHA). NHA express TLR3, TLR4, and TLR5. TLR3 ligation induced the strongest proinflammatory polarizing response, characterized by generation of high levels of TNF-α, IL-6, and IL-8. HIV-1 increased the transient production of key inflammatory mediators, and exposure to LPS of HIV-1-infected cells increased significantly the cytokine secretion. We confirmed that it is necessary viral gene expression from the moment of pretreatment with antiretrovirals inhibited totally HIV-1-induced TLR response. The higher response to LPS from HIV-1-infected cells did not correlate with TLR4 or MyD88 increased expression. LPS responsiveness of infected cells parallels MHC class II expression, but not CD14. HIV-1-infected NHA present increased sensitivity to the proinflammatory effects of LPS. If this phenomenon occurs *in vivo*, it will contribute to the immunopathogenesis of this disease and may ultimately offer novel targets for immunomodulatory therapy.

Human immunodeficiency virus-1 (HIV-1) enters the brain in the early stages of infection resulting in various clinical and pathological abnormalities. The hallmarks of HIV-1 associated neuropathology include brain atrophy, white matter gliosis, and neuronal cell loss^1^,^2^. This neurological damage, especially gliosis and inflammation has been found to correlate with increased production of proinflammatory cytokines and chemokines^3^,^4^,^5^, and can be caused by viral proteins, inflammatory cytokines resulting from systemic infection that cross the blood-brain barrier (BBB) or by toxic factors secreted in the brain by virus-infected or activated cells (reviewed by^6^).

There is growing interest in the potential role of astrocytes in HIV-1-mediated neuropathogenesis. Astrocytes control the homeostasis within the CNS and perform functions mainly associated with regulation of neuronal survey and signaling (reviewed in^7^,^8^). This, associated with the fact that astrocytes are the predominant cells into the brain, supposes an important factor to consider.

Toll-like receptors (TLR) play pivotal roles in the recognition of pathogen-specific patterns and the subsequent initiation of innate and adaptive immune responses^9^. It has been described several effects after TLR stimulation in brain cells, mainly in microglia^10^,^11^. However, the effects of TLR stimulation on human primary astrocytes remain largely unknown. TLR activation is a principal contributor to maintained glial activation, cytokines production and neuronal damage during viral CNS infections; however, insufficient activation results in both inadequate protection during the first days of infection, and in inefficient activation of the adaptive immune system leading to disease or death. It is important to achieve an appropriate balance to fight against viral infections but without the overstimulation of innate responses.

On the other hand, MHC class II molecules can enhance TLR-mediated responses playing a key role in innate immunity^12^. In addition, it has been described that the cooperation of CD14 and TLR4 is required for the molecular and cellular effects of LPS^13^,^14^. Several prior studies have looked at TLR expression in astrocytes and have shown inconsistent findings of the relation between TLR expression and HIV-1 infection. The inconsistencies are likely due to the heterogeneous nature of astrocytes, different sources of astrocytes, and the difficulty in astrocyte infection by cell-free HIV-1.

The current study addressed the TLR expression and function in human astrocytes at basal conditions and following HIV-1 infection.

Our results clearly demonstrate that human astrocytes express TLR3, TLR4, and TLR5. In spite of greater expression of TLR4, the cells respond mainly to TLR3 and TLR5 ligation. Both IL-6 and IL-8 are significantly induced by HIV-1, and HIV-1-infected cells presented an elevated response to LPS, in parallel with MHC class II increased expression. These effects may translate directly in a rise of function playing a role in HIV-1 disease progression.

## Results

### Profile of TLR expression in NHA

**Figure 1:**
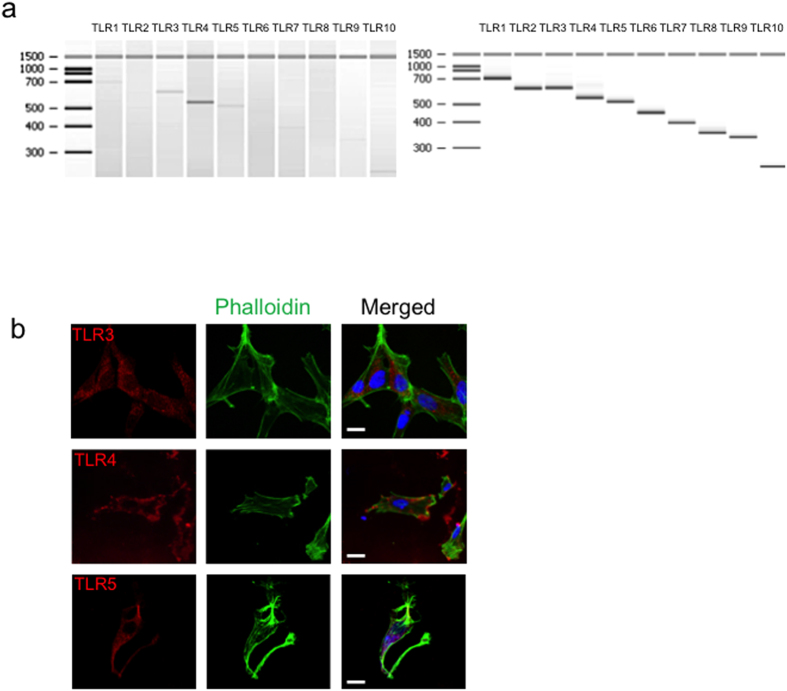
TLR expression in NHA cells under basal conditions. (**a**) mRNA expression of TLR1-10 at basal conditions of NHA (left) and positive controls provided with the kit (right). (**b**) Confocal microscopy for detection of TLR3, TLR4, and TLR5. For surface staining, cells were incubated on ice with TLR antibodies followed by cy5-conjugated F(ab)2 goat anti-rabbit IgG. For intracellular staining cells were fixed, permeabilized with saponin and then stained. FITC- phalloidin and DAPI were also used to label the culture, targeting filamentous actin and nuclear DNA, respectively. Bar scale: 10 μM.

To characterize the expression of TLR 1-10 in normal human astrocytes, NHA cells, we examined the cells under basal conditions. We performed RT-PCR and categorized the range of TLR mRNA expressions by using Bioanalyzer technology. NHA basally express mRNA for TLR3, TLR4, and TLR5 (Fig. 1a), whereas the expression of the rest of TLR was lack compared with the positive controls. To further confirm the expression of TLR and elucidate their subcellular localization, we performed confocal microscopy (Fig. 1b, Supplementary Fig. 1). The cells were also stained with FITC-phalloidin and DAPI, which target F-actin and DNA, respectively. In order to confirm that the fluorescence labeling is due to the binding of the primary antibody to its target, isotype antibodies were used as control for staining (data not shown).

### Functional response of NHA to different TLR ligands

**Figure 2:**
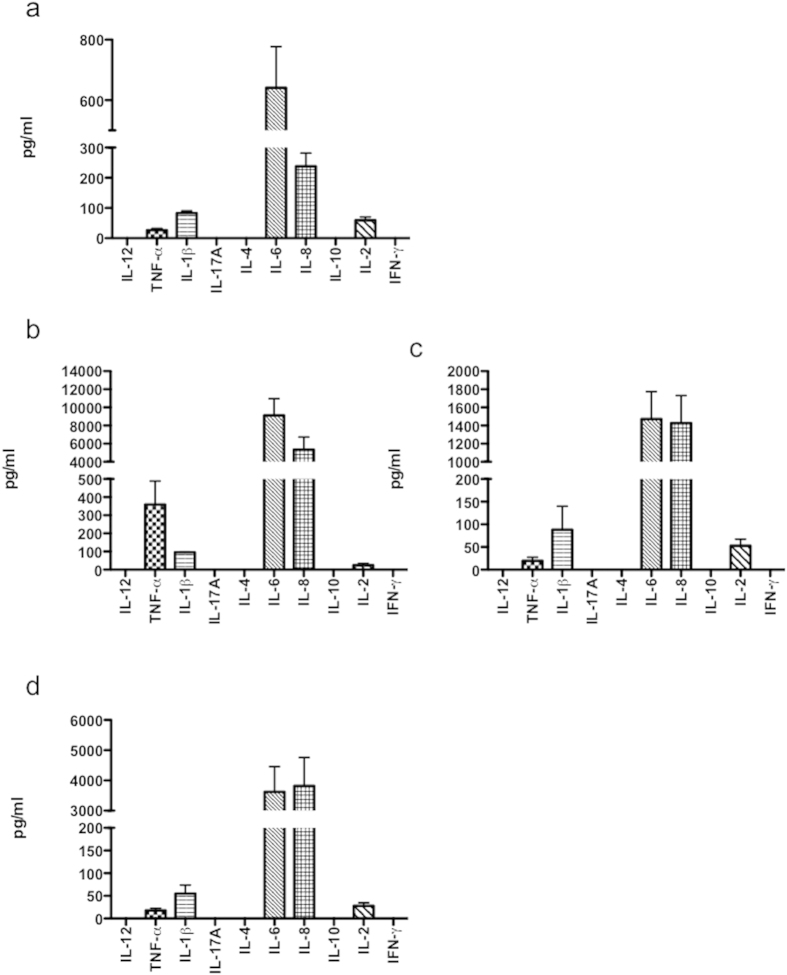
Ligation of TLR induces divergent proinflammatory cytokine secretion by NHA. Cells were untreated (**a**) or treated with poly (I:C) (1 μg/ml) (**b**); LPS (10 μg/ml) (**c**); and flagellin (1 μg/ml) (**d**) for 24 h, and culture supernatants tested by ELISA, as described in M&M. Graphs show mean ± S.E.M. of duplicate samples from four independent experiments.

To elucidate whether the expressed TLR were functional proteins, the cells were incubated with specific ligands as follows; TLR3, poly (I:C) (1 μg/ml), TLR4, LPS (10 μg/ml), and TLR5, flagellin (1 μg/ml). After 48 h pathogen-associated molecular pattern (PAMP)-exposure, conditioned medium was collected (3 inserts per treatment), pooled and screened for the expression of several cytokines by using the commercial kit Th1/Th2 diaplex. At basal conditions, NHA secrete detectable levels of IL-8, and mainly of IL-6 (Fig. 2a). Following TLR3 stimulation cells expressed TNF-α, IL-6 and IL-8 at levels that were 13-fold, 14-fold and 22-fold higher than those in controls, respectively (Fig. 2b). The means of IL-6 and IL-8 productions by LPS stimulated-cells was 2-fold and 6-fold higher than untreated controls, respectively (Fig. 2c). Following flagellin treatment, IL-6 and IL-8 protein concentrations were higher compared with untreated controls (5-fold and 16-fold, respectively) (Fig. 2d).

It is interesting to note that under basal conditions, cells secreted very low levels of IL-1β and IL-2, and there were not differences in their productions under any treatment. As shown in Fig. 2, we failed to detect IFN-γ, IL-17A, IL-10, IL-4 or IL-12.

Summing up, TLR3 ligation was the main stimulus for the production of cytokines. These findings indicate that, in the NHA cells, the level of TLR expression does not correlate with a maximum functional response.

It is possible that stimulation of one or more TLR could result in a differential secretion of cytokines not measured in this study. Another possibility is that a secondary molecule and signal may be needed for TLR response in NHA.

### Cytokine production pattern by HIV-1-infected NHA

**Figure 3:**
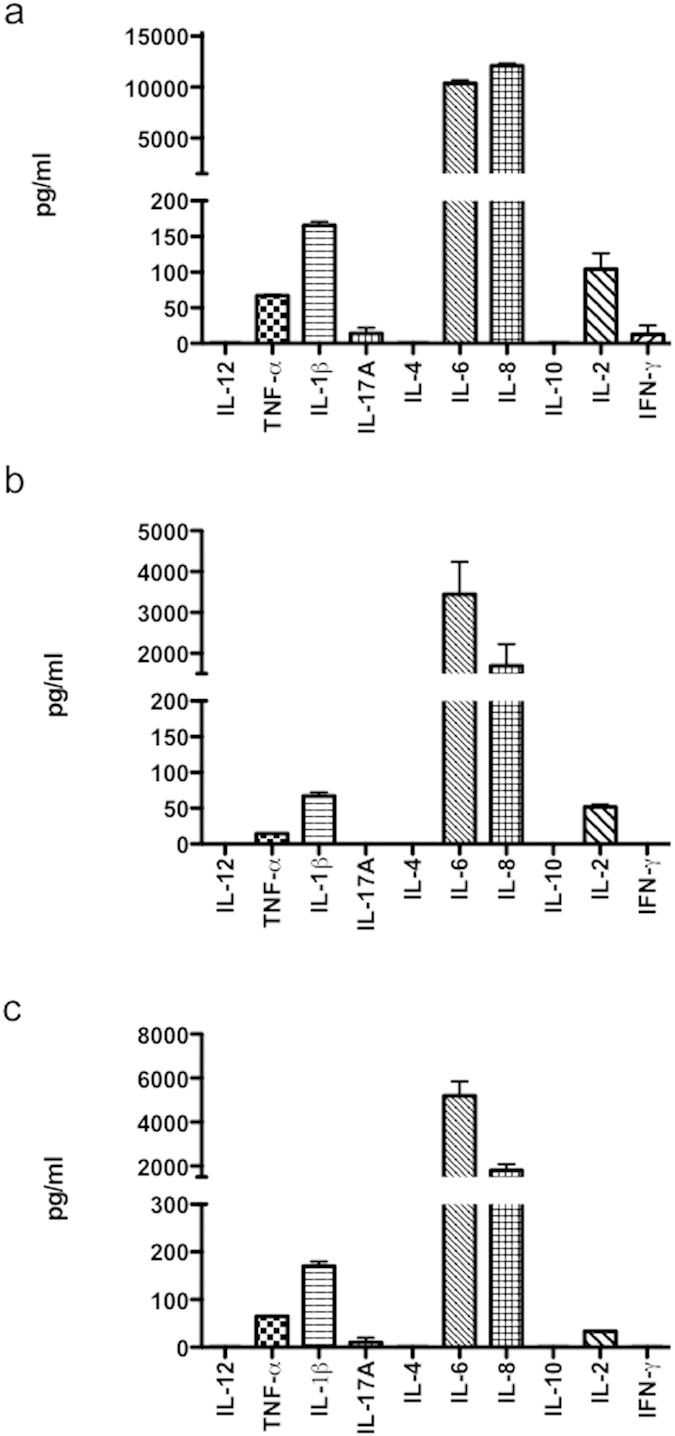
Cytokine production pattern by HIV-1-infected NHA cells. Cytokine concentrations were measured in cell culture supernatants from either HIV-1_NL4-3_ (X4) (**a**), HIV-1_Bal_ (R5) (**b**) or HIV-1gp120 treated cells. Graphs show mean ± S.E.M. of duplicate samples from four independent experiments.

Previous studies have shown that viral envelope protein gp120 can induce a range of neuroinflammatory products in primary astrocytes in culture^15^,^16^, as well as HIV-1 in transformed cell lines^17^,^18^. To investigate the cytokine profile in NHA cells after HIV-1 or gp120 treatment, culture supernatants from treated cells were analyzed 48 h after culture, with either HIV-1gp120 protein, R5 HIV-1_Bal_, or X4 HIV-1_NL4-3_ isolates. HIV-1_NL4-3_ significantly up-regulated the production of IL-6 (16-fold), and to a lesser extent of TNF-α, IL-1β, and IL-2 (Fig. 3a). Notably, X4-infected cells produced IL-8 levels 50-fold higher than that in control cells. The production of IL-6 and IL-8 by R5-infected cells was lower than that produced by X4-infected cells but higher than in controls (Fig. 3b). In addition, HIV-1gp120 protein induced a response significantly lower than that of infected cells (Fig. 3c), indicating that not only the contact with the cell surface stimulates cytokine synthesis.

As only IL-6 and IL-8 were produced at a significant level, we choose these two cytokines for future analysis.

**Figure 4:**
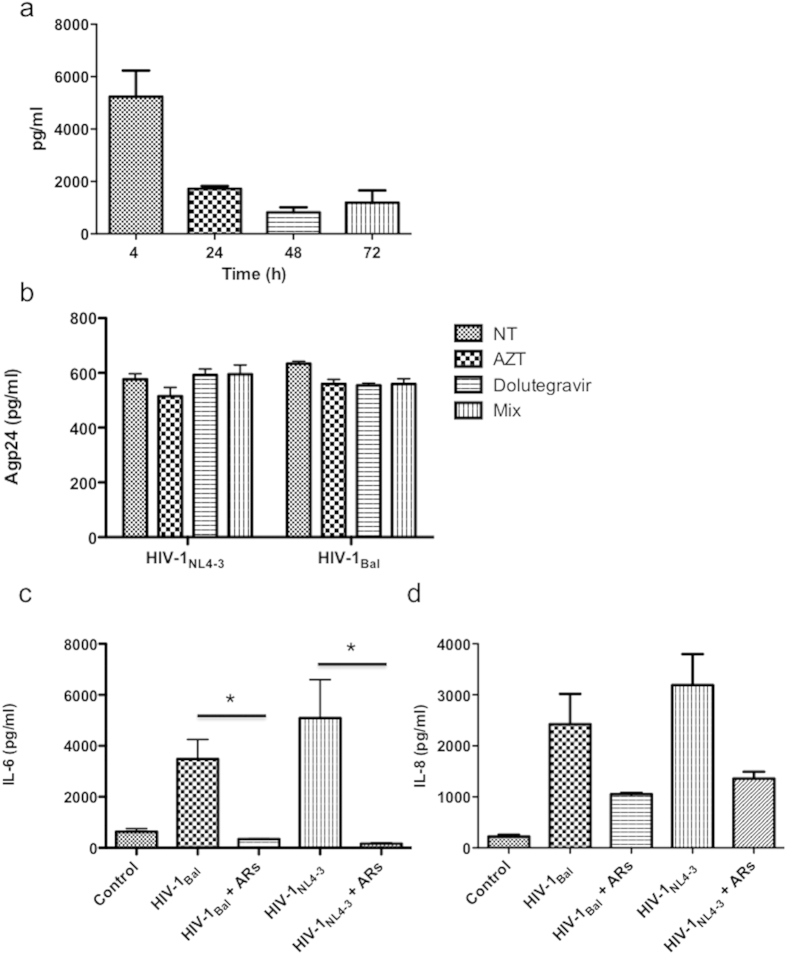
Determination of p24 protein levels in cell cultures. Levels of HIV-1 p24 protein were determined in cell lysates (**a**) at the indicated times, or in the supernatants at 72 h after infection (**b**). (**c**,**d**) Cells were incubated with AZT plus dolutegravir (5 μM) 1 h before infection with R5 and X4 HIV-1 isolates. IL-6 (**c**) and IL-8 (**d**) productions were determined in the supernatants after 48 h of culture. *p ≤ 0.05.

HIV-1 replication of human primary astrocytes is mostly inefficient and restricted. To evaluate whether the effects of HIV-1 on TLR activation resulted from HIV-1 interaction with the cell surface or from HIV-1 gene expression following infection, we monitored HIV-1 entry and replication by measuring p24 protein levels in the cell lysates and in the supernatants of infected cells, respectively. Therefore, cell-associated virus was confirmed by measuring p24 levels in the cell lysates from day zero to 72 h. Levels of intracellular p24 after 4 h of culture were high (Fig. 4a), but these levels decreased over the time indicating a possible endosomal degradation of virus particles previously described^19^. In our conditions, we could not detect an increase over the time of p24 protein in the supernatants, and more important the antiretrovirals (ARs) used did not reduce p24 protein levels (Fig. 4b). Surprisingly, the combination of AZT (5 μM) and dolutegravir (5 μM), RT and integrase inhibitors respectively, inhibited significantly IL-6 secretion induced by HIV-1 either with X4 or R5 viruses (Fig. 4c) (*p < 0.05). Similarly, the IL-8 levels in the cell culture supernatants were reduced by ARs treatment before HIV-1 infection but without significance (Fig. 4d). In summary, HIV-1 entries in primary astrocytes and albeit its inefficient replication, it is necessary gene viral expression to increase cytokine production.

### HIV-1 increases TLR-dependent cytokine production

**Figure 5:**
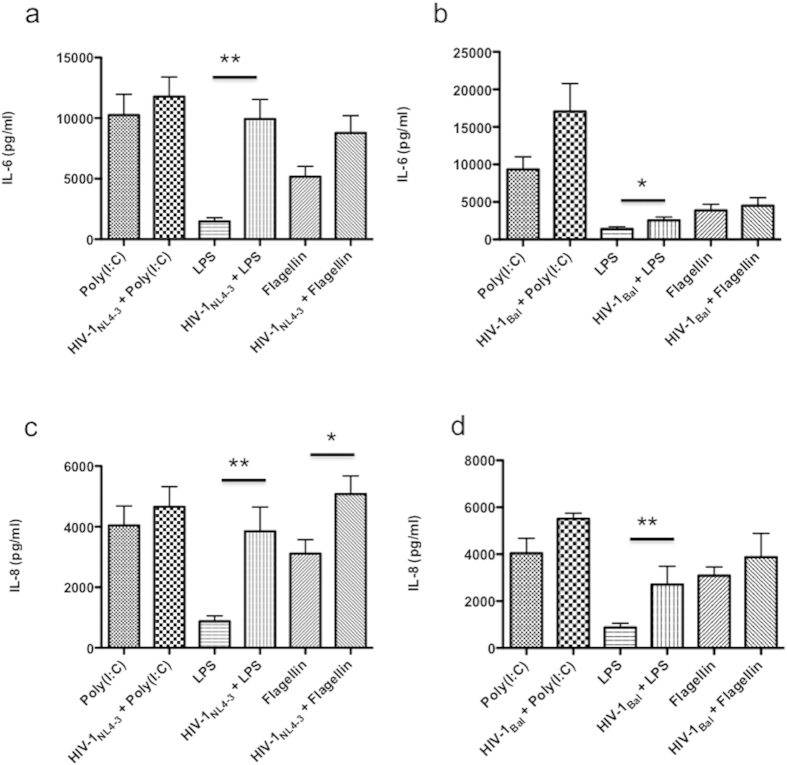
HIV-1 increases TLR responses in NHA. Cells were cultured in the presence of HIV-1_NL4-3_ (X4) or HIV-1_Bal_ (R5) for 72 h before stimulation with poly (I:C), LPS or flagellin, and incubated for 24 h more at 37 °C and 5% CO_2_. IL-8 (left) and IL-6 (right) productions are shown for R5 (**a**,**b**), or X4 (**c**,**d**) isolates. Graphs show mean ± S.E.M. of duplicate samples from four independent experiments. Significant differences *p ≤ 0.05 **p ≤ 0.01.

To investigate whether HIV-1-infection produced altered functional responses of TLR in NHA, the production of IL-6 and IL-8 in response to TLR ligands was analyzed after HIV-1 infection with either X4 or R5 viruses. The expression levels of either IL-6 or IL-8 proteins were not altered in HIV-1-infected cells following TLR3 specific ligation compared with that in untreated controls (Fig. 5). The most incremented response of infected cells either with X4 or R5 viruses was following LPS engagement (Fig. 5a,b). Thus, X4-infected cells secreted significantly higher IL-6 and IL-8 levels after TLR4 ligation than uninfected cells. Also, R5-infected cells secreted higher levels of IL-6, and IL-8 following stimulation with LPS (Fig. 5c,d). Only X4-infected cells presented a potentiated response to flagellin (Fig. 5c). All these results suggest that HIV-1 can alter mainly TLR4 responses.

### HIV-1 up-regulates TLR5 expression in NHA

**Figure 6:**
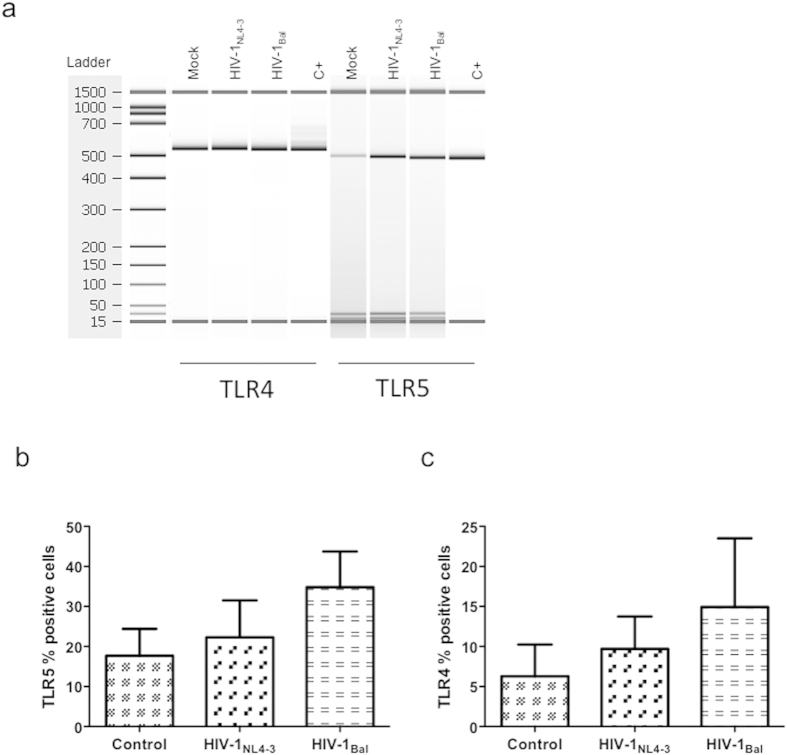
Regulation of TLR expression by HIV-1. NHA were infected with HIV-1_NL4-3_ (X4) or HIV-1_Bal_ (R5), and 48 h later the PCR was used to assay regulation of expression at the RNA level for the indicated TLR (**a**). A representative experiment of three is shown. Surface expression of TLR5 (**b**) and TLR4 (**c**) in HIV-1-infected either with X4 or R5 isolates and control cells. The mean values (mean ± S.E.M.) of three independent experiments are shown.

To determine whether the increased response of infected cells to TLR ligation were due to an alteration of TLR expression, we examined if NHA regulated the expression of individual TLR consequent to *in vitro* HIV-1 infection. PCR analysis revealed that both isolates used altered TLR5 expression, meanwhile no variation of TLR4 levels was found (Fig. 6a). These results were confirmed by flow cytometry, indicating that the up-regulation of TLR5 mRNA level correlated with the HIV-1-induced increase of this protein (Fig. 6b). No significant change of basal levels of expression of TLR4 protein was detected by flow cytometry (Fig. 6c).

Since HIV-1 can increase the expression of TLR5, this could be, at least partially, one of the mechanisms of increased response of infected cells to flagellin. However, as no alteration of TLR4 expression was found, it is possible that other molecules or mechanisms could be involved in the higher specific response to LPS from HIV-1-infected cells.

### HIV-1 does not modify TLR adapter molecules expression

**Figure 7:**
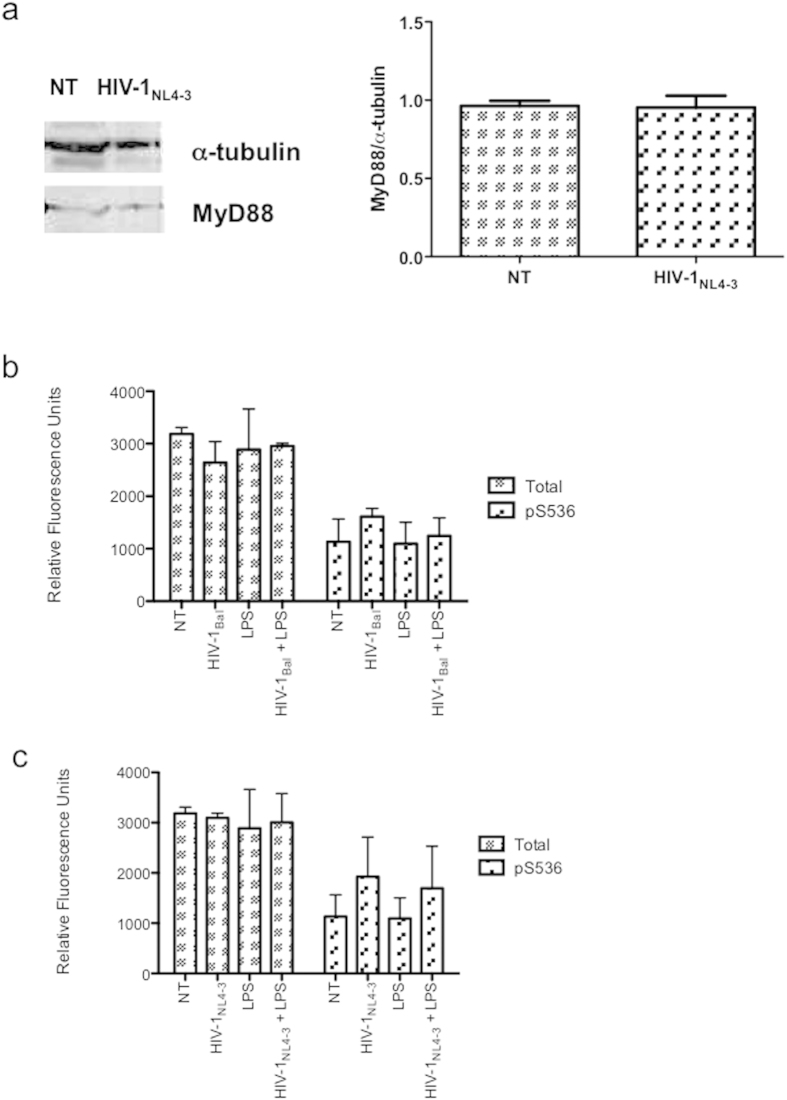
HIV-1_NL4-3_ does not alter either the MyD88 levels or NF-ĸB activation. (**a**) NHA cells were infected with HIV-1_NL4-3_, and 24 h later MyD88 protein levels were determined by western blot. To convert band intensity into a quantitative measurement, the blot was analyzed densitometrically (right). Data present the fold induction relative to control cells. (**b**,**c**) NF-ĸB p65 phosphorylation at Ser536 was determined by ELISA after 20 min of infection with either R5 (**b**) or X4 (**c**) viruses. The mean values (mean ± S.E.M.) of three independent experiments are shown.

Since HIV-1 did not alter TLR4 expression but increased the response to LPS, we wondered whether the subcellular localization of adapter molecules could have a dramatic impact on response to TLR4 ligand. Clearly, TLR4 activates both a common MyD88-dependent pathway and a MyD88-independent TRIF-dependent pathway. To study the possibility of an alteration of levels of MyD88 protein by HIV-1, we performed western blot assays. Our results show that 48 h exposure to HIV-1 does not alter the expression of this protein (Fig. 7a). Because MyD88-dependent signaling cascades can lead to activation of NF-ĸB, we examined whether the HIV-1-dependent increase of cell function extends to the regulation of NF-ĸB activity. To quantify the effect that HIV-1 alone or in combination with LPS had on NF-κB signaling, we investigated the total protein levels and selected the post-translational modification of NF-κB p65 in total cell lysates by ELISA. p65 NF-κB plays a major role in inflammatory cytokine and chemokine production upon TLR ligand stimulation. Total protein and phosphorylated p65 at serine residues 536 were detected in the lysates of untreated and infected astrocytes under different treatments. Although higher levels of Ser536-P-p65 were detected in the lysates of HIV-1-infected cells after 20 min of culture compared with controls, there were not significant differences between groups independently of the tropism of the HIV-1 used (Fig. 7b,c). Furthermore, HIV-induced Ser536-P-p65 was not altered in the presence of LPS.

Taken together, these data indicate that the increased responsiveness of infected cells to LPS is not for modification on TLR4, MyD88 or p65 protein expression.

### LPS responsiveness parallels MHC class II expression, but not CD14 in NHA

**Figure 8:**
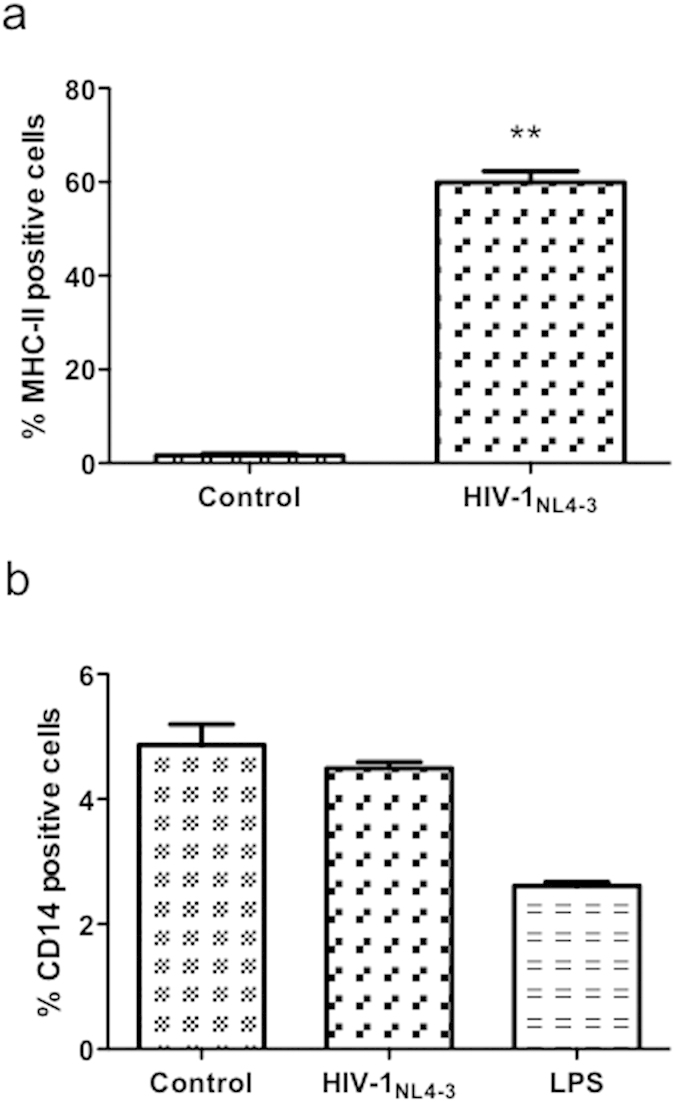
Analysis of MHC class II/CD14 expression. (**a**) Values of % of MHC class II positive cells in the NT and infected cells of four different experiments are shown. (**b**) Surface expression of CD14. After treatments, cells were stained with anti-CD14 and evaluated by flow cytometry. The mean of % of positive cells (mean ± S.E.M.) of three independent experiments is shown. Significant differences were labeled **p ≤ 0.01.

It has been previously described that THP-1, MHC class II positive and CD14 negative monocytic cell human lines stimulated with LPS resulted in the secretion of TNF-α and IL-8. In contrast, THP-1.6, a variant of THP-1 expressing low levels of MHC class II molecules, reduced TNF-α or IL-8 secretion upon stimulation with LPS^20^. In addition to TLR4, the receptor CD14 is required for the molecular and cellular effects of LPS in circulating monocytes^21^. So, we explored the possible involvement of MHC class II and CD14 proteins in the increased response to LPS by HIV-1-infected cells. NHA were exposed to HIV-1 for 24 h, and MHC class II and CD14 expression was evaluated by flow cytometry. HIV-1 up-regulated the levels of MHC class II protein (Fig. 8a) (**p ≤ 0.01), but did not alter the CD14 levels over control (Fig. 8b).

### TLR4 ligation of NHA does not modulate migration across *in vitro* model

**Figure 9:**
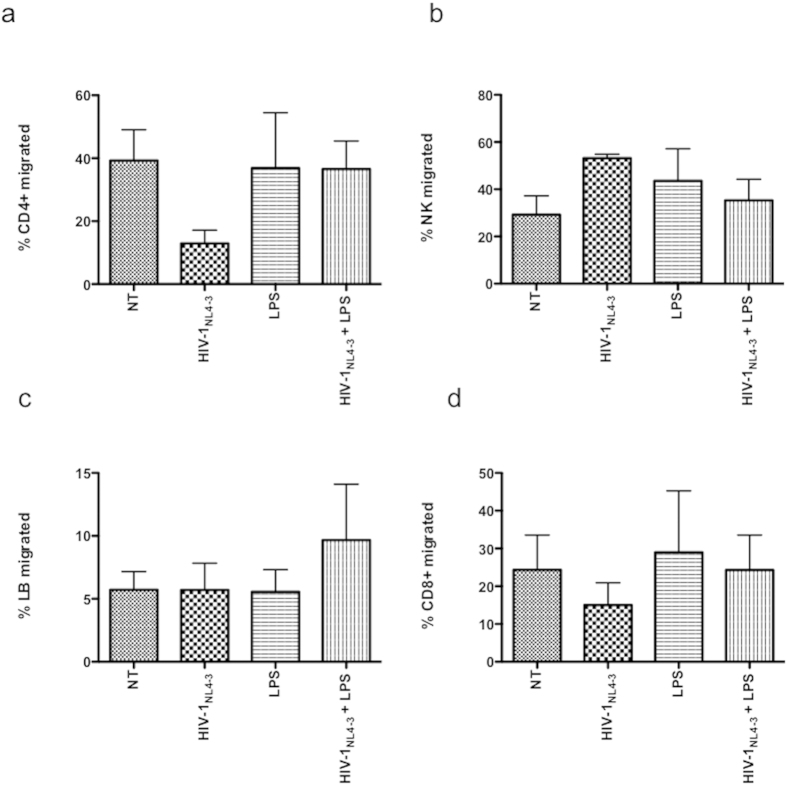
Migration of PBL toward supernatants from HIV-1-infected NHA. IL-2-activated PBL were transferred to the upper chamber of the transwells and allowed to migrate in response to astrocytes under different conditions in the lower chamber for 4 h. Migrated cells were counted and stained with anti-CD3-PE, anti-CD19-ECD, anti-CD56-PC5, anti-CD4-PC7 and anti-CD8-FITC. The number of migrated CD4^+^T (**a**), NK cells (**b**), LB (**c**), and CD8^+^T (**d**) was counted and expressed as a percentage of the total migrated cells. The mean values (mean ± S.E.M.) of five independent experiments are shown.

HIV-1 and secreted viral factors induce chemotaxis and migration of infected leukocytes across the BBB^22^,^23^. To determine the functional significance of HIV-1-LPS-induced up-regulation of IL-6 and IL-8 expression, transwell migration assays were set up using PBL. After 4 h of the experiment, PBL populations were collected and tested by flow cytometry. Within the time frame of the experiment, minimal migration of PBL was observed in response to supernatants from unstimulated astrocytes. Individually, supernatants from HIV-1-infected NHA decreased CD4^+^T cells and CD8^+^ T cells (Fig. 9a,d) and increased NK migration (Fig. 9b) across a model of transwell described in material and methods, although without significance perhaps due to the low number of experiments analyzed. In addition, we observed an increase of lymphocytes B (LB) migrated population after LPS treatment of HIV-1-infected cells (Fig. 9c). Supernatants from astrocytes treated with LPS alone did not induce significant variation of migrated populations tested under any condition investigated.

## Discussion

There exist several lines of evidence about HIV-1 infection of astrocytes and its contribution to the overall inflammatory response in the brain of infected people (reviewed in^24^,^25^). Earlier reports have shown that in infected brain tissues, up to 25% of astrocytes are positive to HIV-1 DNA^26^,^27^. It has been previously described that HIV-1 *in vitro* infection of astrocytes leads to an initial transient, short-term burst of virus production that is followed by a restriction with the production of few viral progeny or latency^28^. Also, some studies have suggested intracellular restrictions, with the presence of efficient early viral transcripts, but low levels of late transcripts responsible for structural proteins^29^. We and others have demonstrated that productive infection can be reestablished following stimulation with cytokines such as TNF-α and IL-1β^30^,^31^ or bryostatin^32^, but HIV-1 levels still consistently remain lower than those documented in cells, such as microglia, and monocytes.

Astrocytes express a more limited TLR repertoire, probably because they are not classical immune cells based on their neuroectodermal origin, but can contribute to inflammation if necessary. In the present study, we describe the expression of TLR3, TLR4 and TLR5 at mRNA and protein levels in human primary astrocytes. Following stimulation with the appropriate ligands, NHA present different pattern of cytokines. Therefore, stimulation with poly (I:C) induced TNF-α secretion and treatment with poly (I:C), LPS or flagellin, increased IL-6 and IL-8 productions. In our model, TLR3 ligation induced the higher response compared with that in control cells albeit its lower expression. Notably, typical proinflammatory cytokine, such as IL-1β was not produced at high levels. This result is in line with a previous study that shows that IL-1β is undetectable at both mRNA and protein levels in non-stimulated or cytokine-stimulated cultured human astrocytes^33^, but in contrast with earlier findings that show that TLR2 and TLR4 activation induces high production of the proinflammatory cytokines IL-1β and TNF-α. Observed discrepancies may be a result of differences in experimental design (*in vivo* versus *in vitro*), the presence or absence of microglial cells, cells selected (primary cells versus cell lines), or treatment duration.

Viral-induced inflammation and dysregulation of cytokine expression play a major role in the pathogenesis of HIV-1 infection and disease progression^34^,^35^. Elevated levels of IL-6 and IL-8 were found in the cerebrospinal fluid of HIV-1 infected patients, and it has been suggested a possible link between cytokine levels and neurological complications^34^. HIV-1 proteins as gp120, Tat or Nef has been mostly involved in neuroinflammation as these proteins have been shown to increase the production of a variety of proinflammatory cytokines as IL-6^36^,^37^, IL-1β^38^, IL-8^36^, and MCP-1^39^.

In line with this, we found that HIV-1-infected cells, mainly with X4 virus, secreted higher levels of IL-6 and IL-8 compared with that produced by control cells. Notably, X4-infected astrocytes produced IL-8 levels 50-fold higher than uninfected, and 16-fold higher levels of IL-6. It is interesting to note the lower production of cytokines following R5 infection.

When the primary astrocytes were treated with HIV-1gp120 protein, there was an important induction of IL-6 and IL-8 but significantly lesser than in HIV-1 exposed cells, indicating that this response is not only a surface-mediated phenomenon. When ARs were used to investigate the effects on HIV-1 replication and cytokine production, we found that there is no effect on p24 secretion upon treatment even after 48 h post-treatment. Clarke *et al.* presented similar data, showing that the detection of HIV-1 DNA in a glioblastoma cell line was also found to be independent of pre-treatment with AZT, and 3TC. The authors proposed a non-replicative mode of HIV-1 persistence and transmission^19^. More recently, it has been determined that 3TC, d4T and ZDV may not effectively target astrocyte infection *in vivo*^40^. Strikingly, although the ARs selected in this work did not interfere with viral replication, the pretreatment with them inhibited largely the secretion of IL6 and IL8 induced by HIV-1 especially by X4 isolate, indicating that the altered cytokine response of HIV-infected astrocytes is an intracellular mediated phenomenon and viral gene expression following entry is necessary. In this regard, previously, other groups have proposed that some antiretrovirals, as AZT, could interfere directly with protein production in glial cells independently from their antiviral activity^41^.

Sun *et al.* described that HIV-1 infection increased the responsiveness of mice astrocytes following LPS ligation. In this work, the authors show that LPS stimulated astrocytes secreted CCL2/MCP-1 and induced the migration of monocytes to the brain^42^. In line with this, here we demonstrate that LPS induce HIV-1-infected human primary astrocytes to produce increased levels of IL-6 and IL-8.

Following interaction with ligand, a highly conserved cytoplasmic Toll-IL-1R domain is engaged that triggers the activation of the NF-ĸB, JNK and p38 MAPK signaling pathways via the recruitment of MyD88^43^. Several studies demonstrated that HIV-1 infection interfered with some of these intracellular pathways^44^; however, in our hands, HIV-1 had no effect neither on expression of MyD88 nor in the levels of Ser536-P-p65. Indeed, HIV-1 infection followed by activation with LPS did not result in a variation of the previous levels. Although preliminary, these results could exclude these proteins on increased responsiveness to LPS of the infected cells.

CD14 and TLR4 are required for the molecular and cellular effects of LPS in cells as circulating monocytes^13^,^14^. HIV-1 did increase neither TLR4 nor CD14 levels that let us explain the increased response to LPS. All these data suggest that increased sensitivity of HIV-1-infected cells to LPS is not dependent on a disproportionate cell population expressing TLR4 or CD14.

MHC class II molecules play a crucial role a key role in innate immunity by cooperating with TLR response in inducing an innate immune response^12^. Recently, it has been described that intracellular MHC class II molecules can act as adaptors promoting full activation of TLR-triggered innate immune response^45^. While microglia express MHC class II readily upon activation *in vivo* and *in vitro*, astrocyte MHC class II expression occurs only during prolonged inflammation *in vivo* or *in vitro* under stimulation by IFN-γ^46^. It could be likely that the increase in MHC II is driven by IFN, but we discarded this idea since primary astrocytes did not secrete detectable levels of this protein after culture with HIV-1. Piani *et al.* demonstrated that secretion of pro-inflammatory cytokines in response to LPS is modified by MHC class II molecules^20^. In this work, the authors propose that MHC class II participate both in the antigen-unspecific, inflammatory response to microbes and in the antigen-specific adaptive immune response. It is possible that HIV-1 virus increases MHC class II expression and by the way the response to LPS. Whether is necessary the direct binding of LPS to MHC class II for the contribution of these molecules to LPS responsiveness in infected cells, or whether other mechanisms are responsible for this effect is currently investigated.

In addition, although the majority of inflammatory pathology in adults is associated with microglia/macrophages, it is likely that the effects noted here contribute synergistically to the inflammatory mediation by the principally infected cell type, or even help to amplify the reaction of macrophages. Future studies regarding this issue will be performed in the future.

It is well determined that HIV-1 infected individuals present increased circulating LPS levels due to microbial translocation across a compromised mucosa barrier. If we compare the cytokine production between HIV-1-infected cells stimulated or not with LPS, we find that stimulated cells produce IL-6 more potently than unstimulated ones (**p ≤ 0.01) (Supplementary Fig. 2), indicating the importance of exposure to PAMPs in HIV-1-infected patients. Entry and recognition of PAMPs to their specific receptors, as TLR, could initiate a very important cytokine production increasing the risk of cell activation and neuronal death.

## Conclusions

We provide evidence that human primary astrocytes can function as a part of the innate immune response, sensing PAMPs. More important, they can mount an increased inflammatory signal upon exposure to any of the studied TLR ligands or HIV-1, and indeed infected cells present special sensitivity to LPS. Further studies are warranted to confirm our observations; however, if increased TLR5 expression in primary astrocytes, as well as increased inflammatory response to TLR4 by HIV-1-infected cells occurs *in vivo*, it can contribute to the neuropathogenesis of the disease and may ultimately offer novel targets for brain therapy.

## Materials and Methods

### Cell cultures

Normal human astrocytes (NHA) isolated from the cerebrums of 5-month-old human fetuses were purchased from Cambrex (CC-2565, NHA-normal human astrocytes; Walkersville, MD, USA) were cultured according to previously described^47^. To avoid possible lot-specific cell responses, several vials of astrocytes derived from different isolations (different lots) were purchased. Cultures were harvested using Reagent- PackTM (Cambrex) including trypsin neutralizing solution, trypsin/EDTA, and HEPES buffered saline solution.

Human peripheral blood lymphocytes (PBL) were isolated from heparinized blood of healthy donors using density centrifugation over Ficoll-Hypaque (GE Healthcare, Little Chalfont, Buckinghamshire, United Kingdom) as previously described^48^, and activated with 2 μg/ml phytohemagglutinin, (PHA) and 60 U/ml interleukin-2 (IL-2). MT-2 (T cell leukemia) cells were routinely grown in RPMI 1640 (Biochrom KG Seromed, Berlin, Germany) supplemented with 10% heat-inactivated fetal calf serum (FCS), 1% penicillin/streptomycin, and 2 mM l-glutamine (ICN Pharmaceuticals, Costa Mesa, CA) at 37 °C in a humidified atmosphere of 5% CO_2_.

AZT (as RT inhibitor) and dolutegravir (as integrase inhibitor) were purchased from Sigma (St. Louis, MO, USA).

### Virus stock production and cell treatments

X4 HIV-1_NL4-3_, and R5 HIV-1_Bal_ isolates stocks were prepared by infecting MT-2 cells and p24Gag antigen was determined by ELISA (Innotest HIV-1 antigen mAb; Innogenetic, Ghent, Belgium) as described earlier^48^,^49^ as a marker of HIV-1 infection. HIV-1 stock was then used to infect NHA cells. Briefly, 2 × 10^5^ NHA cells were infected with both viruses containing (200 ng HIV-1 p24Gag/10^6^ cells). After 2 h incubation, the cells were treated with trypsin to remove residual extracellular virions bound to the external surface of the cells, and resuspended in fresh medium and maintained for the indicated times for each condition as previously described^50^,^51^. In selected experiments, cells lysates were performed with phosphate buffered saline (PBS) 2% Triton X-100 (Sigma, St. Louis, MO, USA).

### RNA isolation, reverse transcription (RT) and PCR

RNA was extracted by using the RNeasy Mini kit and RNase-free DNase set (Qiagen, Hilden, Germany). One microgram of RNA was used for each cDNA synthesis (GoScript Reverse Transcription System; Promega, Madison, WI, USA). All steps were performed by following the manufacturer’s protocol.

PCR was performed with Human TLR RT-Primer Set (InvivoGen, San Diego, CA) that contains a primer pair for all ten human TLR. Each RT-PCR primer pair is provided with a positive control. For more precise quantification, targeted PCR reactions were carried out, and the amplified products were analyzed by automated chip-based microcapillary electrophoresis on an Agilent 2100 Bioanalyzer instrument (Agilent Technologies, Santa Clara, CA) as previously described^52^,^53^.

### Flow cytometric analysis

Cells were fixed and stained with antibodies (Abs) against CD14-PC7 (Beckman-Coulter) and MHC class II (donated by Dr. Sánchez-Madrid) to measure surface expression of both proteins. Isotype controls matched for the concentration of the primary Abs were used for all stainings. After incubation, cells were washed with PBS, collected for analysis by flow cytometry using the EPICS-XL MCL and analyzed using Kaluza software (Kaluza Flow Cytometry, Becton Dickinson).

### Multiple analyte detection

Cells were incubated for 48 h in medium containing selected TLR ligands (all from InvivoGen, San Diego, CA), as follows: TLR3, Poly(inosinic acid):poly(cytidylic acid) (poly (I:C) (PIC)), a synthetic dsRNA (1 μg/ml); TLR4, ultrapure LPS (10 μg/ml); and TLR5, flagellin (1 μg/ml). After incubation, supernatants were collected and analyzed using a DIAplex Human Th1/Th2/Inflammation kit (Diaclone, Gen-Probe). DIAplex Human Th1/Th2/Inflammation is a multiplexed fluorescent bead-based immunoassay for the quantification of multiple human cytokines in serum and culture supernatants by flow cytometry: IFNγ, TNF-α, IL-1β, IL-2, IL-4, IL-6, IL-8, IL-10, IL-12p70 and IL-17A.

### Migration assays

For evaluating lymphocyte migration, experiments were performed in 24-well plates with 5 μm-pore size polyester membrane inserts (Corning Inc.). rhIL-2-activated PBL (10^5^) in 200 μl of RPMI medium were placed in the upper chamber. NHA cells (3 × 10^5^/300 μl) under different treatments for 24 h were seeded on lower chambers. Lower chambers with medium only served as a control for spontaneous migration. After 4 h incubation at 37 °C migrated cells were counted and stained with anti-CD3-PE, anti-CD19-ECD, anti-CD56-PC5, anti-CD4-PC7 and anti-CD8-FITC. The number of migrated T, B and NK cells was counted and expressed as a percentage of the total migrated cells.

### Western blot

Cells were exposed to different stimuli, washed with PBS, and lysed on ice for 10 min in lysis buffer, which is RIPA buffer (50 mM Tris-HCl, pH 7.2, 0.15 M NaCl, 1.0 mM EDTA, 0.1% SDS, 1.0% Triton X-100, 1.0% sodium deoxycholate) freshly supplemented with protease inhibitors. Protein contents were determined using the bicinchoninic acid (BCA) Protein Assay kit (Pierce, Rockford, IL, USA). Samples (30 μg) were separated on 10% SDS polyacrylamide gel and blotted on a polyvinylidene fluoride membrane (Millipore, Bedford, MA, USA) by semidry transference blotting. Membranes were blocked overnight at 4 °C using Rotiblock (Roth, Karlsruhe, Germany) before incubation with an anti-MyD88 (ProSci, CA, USA) as primary antibody. After washing, horseradish peroxidase-conjugated secondary antibody (Amersham, GE Healthcare, UK; 1:10000) was used at 1:5.000 dilution. Proteins bands were detected using the Immun-Star Western C Kit (Bio-Rad Laboratories, Hercules, CA, USA). α-tubulin (Sigma, St. Louis, MO, USA) was used as internal control to validate the amount of protein loaded on the gels. For quantitation, the pixel intensity for each band was determined using the Image/J program.

### Confocal microscopy

Cells were fixed in PBS (pH 7.4) containing 3.7% paraformaldehyde and 0.025% glutaraldehyde for 10 min and permeabilized with PBS 0.1% saponin for 10 min. After two washes, cells were blocked 1% bovine serum albumin (BSA) in PBS for 20–30 min at 25 °C. Samples were then incubated for 1 h at 23 °C with the appropriated antibodies (FITC-labeled phalloidin (Invitrogen, Carlsbad, CA, USA), and different Abs anti-TLR) or isotype-matched control antibodies. After a final washing with PBS, DAPI (1 μg/ml; Alexis Biochemicals) was applied to label nuclei and coverslips were mounted on slides using Fluoromount-G (SouthernBiotech). Imaging was performed using an inverted confocal fluorescence microscope (SP2; Leica Microsystems, Heidelberg, Germany). Separate images were taken in the corresponding channels, and merge images were composed. Image acquisition and data processing for all the samples were performed under the same conditions.

### Measurement of NF-ĸB activity

NF-ĸB activation was measured and quantified by using the NFkappaB p65 pSer536 + Total PhosphoTracer ELISA Kit (Abcam plc, Cambridge, UK).

### Statistical analysis

The nonparametric Wilcoxon signed rank test was used throughout. All statistical analyses were performed with GraphPad Prism 4.0 (GraphPad Software, San Diego, CA). All tests were performed in duplicates and data are means ± S.E.M. Significant differences were labeled *p ≤ 0.05, **p ≤ 0.01, and ***p ≤ 0.001.

## Additional Information

**How to cite this article**: Serramía, M. J. *et al.* HIV-1 increases TLR responses in human primary astrocytes. *Sci. Rep.* **5**, 17887; doi: 10.1038/srep17887 (2015).

## Supplementary Material

Supplementary Information
