# Supplementary material for: HIV-1 increases TLR responses in human primary astrocytes
Source: Sci Rep. 2015 Dec 16;5:17887. doi: 10.1038/srep17887 (PMC4680863; doi:10.1038/srep17887)
Supplement: Supplementary Information [file srep17887-s1.pdf]

HIV-1 increases TLR responses in human primary astrocytes

M[a] Jesús Serramía, M[a] Ángeles Muñoz-Fernández, and Susana Álvarez#

Laboratorio InmunoBiología Molecular, Plataforma de Laboratorio, Hospital General Universitario Gregorio Marañón; Spanish HIV HGM BioBank; Instituto de Investigación Sanitaria Gregorio Marañón, Madrid, Spain; Networking Research Center on Bioengineering, Biomaterials and Nanomedicine (CIBER-BBN), Madrid, Spain.

Supplementary information.

Figure S1. TLR expression in NHA primary human astrocytes under basal conditions. Protein expression of TLR3, TLR4, TLR5 and TLR9 measured by confocal analysis. Phalloidin and DAPI labeling were used in order to visualize the actin cytoskeleton and cell nuclei, respectively. Bar scale: 10 μM.

Figure S2. Cytokine secretion by HIV-1-infected cells stimulated or not with LPS. NHA infected cells were exposed or not to LPS for 48 h and IL-6 (left) and IL-8 (right) levels were measured in the supernatants of the cultures by flow cytometry. Graphs show mean ± S.E.M. of duplicate samples from four independent experiments. Significant differences $^{**}p \leq 0.01$.

Figure S1.

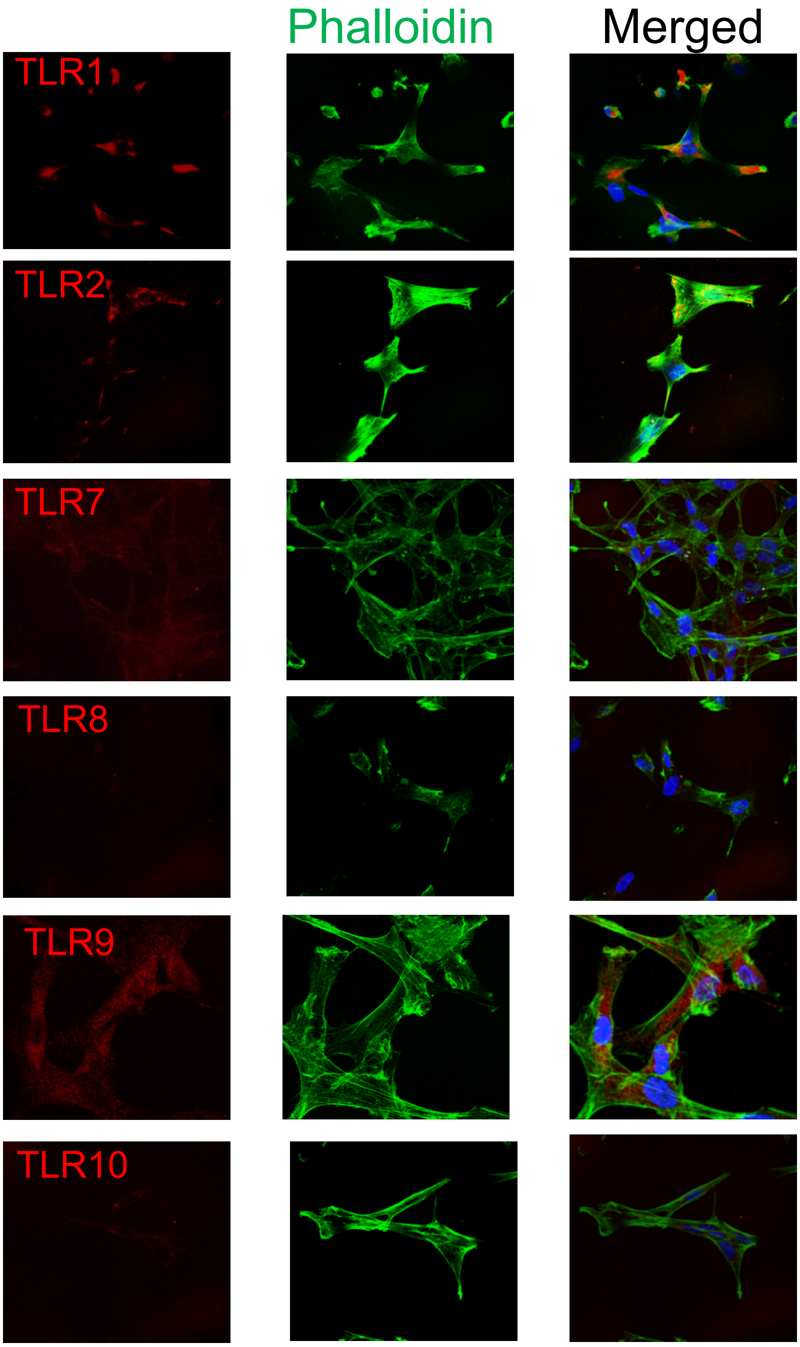

Figure S2.

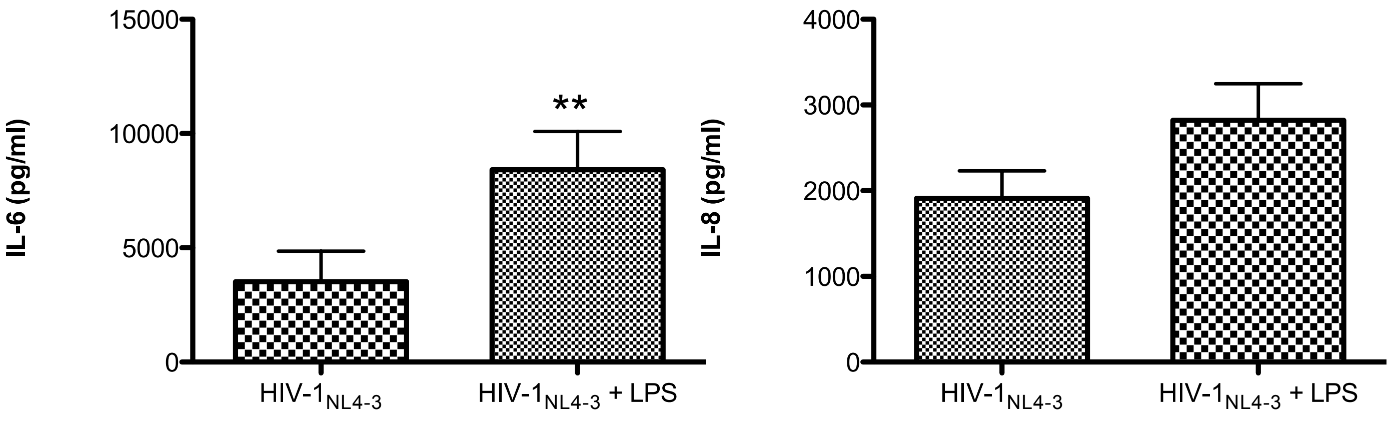

IL-6 (pg/ml)
15000
10000
5000
0
**
HIV-1NL4-3
HIV-1NL4-3 + LPS
IL-8 (pg/ml)
4000
3000
2000
1000
0
HIV-1NL4-3
HIV-1NL4-3 + LPS
